# Evaluation of Cytokine Profile in Canine Malignant Oral Melanoma

**DOI:** 10.3390/vetsci12070627

**Published:** 2025-06-30

**Authors:** Carmen G. Pérez-Santana, Sara E. Cazorla-Rivero, Ana A. Jiménez-Alonso, Francisco Rodríguez-Esparragón, Jesús María González Martín, Ruth Henríquez-Cabrera, Bernardino Clavo-Varas, Enrique Rodríguez Grau-Bassas

**Affiliations:** 1 Instituto Universitario de Sanidad Animal y Seguridad Alimentaria (IUSA), Universidad de Las Palmas de Gran Canaria (ULPGC), 35400 Arucas, Spain; 2 Unidad de Investigación Hospital Universitario de Gran Canaria Dr. Negrín, 35010 Las Palmas de Gran Canaria, Spain; 3 Fundación Canaria Instituto de Investigación Sanitaria de Canarias (FIISC), Hospital Universitario de Gran Canaria Dr. Negrín, 35010 Las Palmas de Gran Canaria, Spain; 4 Universidad de La Laguna, 38200 La Laguna, Spain; 5 CIBER de Enfermedades Respiratorias, Instituto de Salud Carlos III, 28029 Madrid, Spain; 6 Instituto Universitario de Enfermedades Tropicales y Salud Pública de Canarias, Universidad de La Laguna, 38296 La Laguna, Spain; 7 CIBER de Enfermedades Infecciosas (CIBERINFEC), Instituto de Salud Carlos III, 28029 Madrid, Spain; 8 Chronic Pain Unit, Hospital Universitario Dr. Negrín, 35010 Las Palmas de Gran Canaria, Spain; 9 Radiation Oncology Department, Hospital Universitario de Gran Canaria, Dr. Negrín, 35010 Las Palmas de Gran Canaria, Spain

**Keywords:** cancer, dogs, oral melanoma, oncology, cytokine, immunotherapy, therapeutic targets, canine spontaneous tumor model

## Abstract

Ten dogs with oral malignant melanoma were evaluated and treated with surgery, of which four dogs were diagnosed with melanotic melanoma and six were diagnosed with amelanotic melanoma. Serum samples from oral malignant melanoma (OMM) were collected at baseline, the day of the surgery, and every 3–4 months, during which time a clinical examination and chest X-rays were performed. Concentrations of GM-CSF, IFN-γ, IL-2, IL-6, IL-7, IL-8, IL-10, IL-15, IL-18, IP-10, KC-like, MCP-1, and TNFα were quantified. Follow-up samples indicated that after the removal of malignant melanoma, the serum levels of GM-CSF, IFN-γ, MCP-1, IL-18, and IL-2 increased significantly. In contrast, when comparing samples from dogs with OMM to those of patients in remission, the concentrations of IL-7 and MCP-1 were significantly higher in the remission samples than in the OMM samples. Furthermore, when comparing the serum concentrations between the OMM-metastasis samples and those patients in remission, elevated levels of MCP-1 were associated with poorer overall survival due to the development of OMM metastasis. Finally, a comparison of cytokines in the melanotic OMM and amelanotic OMM samples revealed that the amelanotic OMM samples exhibited higher concentrations of IL-6, IL-10, and IL-15 compared to the melanotic OMM samples.

## Simple Summary

Canine models have been recognized as valuable tools for studying various human cancers, including melanoma. Research on carcinogenesis and the development of innovative cancer therapies is progressing rapidly, leading to improved treatment options in veterinary medicine. Despite this progress, the prognostic role of cytokines in canine oncology remains insufficiently investigated. This prospective study reports on 10 cases of oral malignant melanoma (OMM) in dogs that underwent surgical treatment. Among them, four were diagnosed with melanotic and six with amelanotic melanoma. Serum samples were collected at baseline (prior to surgery), on the day of surgery, and subsequently every 3–4 months, accompanied by clinical examinations and thoracic radiographs. Concentrations of GM-CSF, IFN-γ, IL-2, IL-6, IL-7, IL-8, IL-10, IL-15, IL-18, IP-10, KC-like, MCP-1, and TNFα were quantified. Follow-up samples indicated that after the removal of malignant melanoma, the serum levels of GM-CSF, IFN-γ, MCP-1, IL-18, and IL-2 increased significantly. In contrast, when comparing samples from dogs with oral malignant melanoma to those without the disease, concentrations of IL-7 and MCP-1 were significantly higher in the absence of disease samples than in the OMM samples. Furthermore, when comparing serum concentrations between samples from OMM patients with metastasis and those patients in remission, elevated levels of MCP-1 were associated with poorer overall survival due to the development of OMM metastasis. Lastly, a comparison of cytokines in samples from melanotic OMM and amelanotic OMM revealed that amelanotic OMM samples exhibited higher concentrations of IL-6, IL-10, and IL-15 compared to their melanotic counterparts. This study contributes to the evidence that canine models can offer valuable insights that may also translate into more effective and targeted treatments for human melanoma.

## 1. Introduction

Dogs have been suggested as a useful model for several types of human cancer, including melanoma [1,2]. The in-depth characterization of these models will expedite the development and application of new therapeutic strategies. Comparative genomic analyses between human and canine tumors underscore the value of the dog model in advancing knowledge of tumor initiation and progression in humans [3]. These studies have accelerated the identification of clinically relevant treatments for both species. Immunotherapy, in particular, shows promise as a strategy due to its potential for systemic but cancer-specific therapeutic effects [4,5].

Immunotherapy is now recognized as one of the fundamental pillars of human cancer treatment, with clinical importance equivalent to surgery, radiotherapy, and traditional chemotherapy [4,6,7]. The study of carcinogenesis and the development of different cancer therapies is an extremely rapidly advancing area of research [8,9]. New treatment modalities are being developed to provide better veterinary care for canines [10,11].

Immune checkpoint inhibitors (ICIs) are widely used to treat human cancers, and growing evidence suggests that ICIs are promising treatments for canine malignancies [12]. To date, several serum factors have been identified that are predictive of ICI benefit among human cancer patients, including reactive protein (CRP), IL-6, soluble PD-L1, and various cytokines and chemokines [13,14,15]. However, the predictive value of cytokines is still under-investigated in canine cancer. In veterinary medicine, several clinical studies have evaluated the efficacy of immune checkpoint blockade for dogs with cancer [11,12].

Therefore, we aimed to prospectively identify specific cytokine levels or profiles that could serve as biomarkers and provide information either on disease status, tumor presence or absence after surgery, or that would allow distinguishing between metastases and tumor subtypes throughout the follow-up period.

## 2. Materials and Methods

Patient samples. Dogs affected by oral malignant melanoma that were presented at the Veterinary Oncology Service of GICOREC IUSA (Instituto Universitario de Sanidad Animal y Seguridad Alimentaria) of the Universidad de Las Palmas de Gran Canaria (ULPGC, Gran Canaria, Spain) since 2021, with a minimum follow-up of 1 year to 2024, were prospectively considered for this study. The dogs were presented for surgical treatment and were treated according to the Good Clinical Practice guidelines for animal clinical studies and approved by the bioethics committee of ULPGC (OEBA-ULPGC 33/2020R1).

Ten dogs were staged by obtaining a thorough clinical examination, complete blood cell count, serum biochemistry profile, X-rays of the thorax (three views), and total body computed tomography (CT) when indicated. Canine candidates for surgical excision of the primary tumor with or without regional lymphadenectomy and a histopathologically confirmed diagnosis of oral melanoma were included.

A total of 4 mL of blood was obtained from the jugular vein and immediately centrifuged to separate the serum. The samples were collected for the first time from oral malignant melanoma (OMM) dogs on the day of surgery. Subsequent samples were obtained every 4 months, during which time a clinical examination and chest X-rays were performed, until the completion of the 1-year follow-up. Concentrations of granulocyte-macrophage colony-stimulating factor (GM-CSF), interferon γ (IFN-γ), interleukins (IL)-2, IL-6, IL-7, IL-8, IL-10, IL-15, IL-18, interferon gamma-induced protein 10 (IP-10), keratinocyte chemotactic-like (KC-like), monocyte chemotactic protein-1 (MCP-1), and tumor necrosis factor α (TNFα) were quantified with MILLIPLEX Canine Cytokine Magnetic Bead Panel (Merck Millipore) using Luminex technology LABScan3D (XMAP).

Statistical analysis. Results are expressed as median, standard deviation, percentiles, and quartiles. The Shapiro–Wilk test was applied to assess the distribution of the quantitative variables. Pearson’s or Spearman’s coefficients were employed to evaluate the correlations between numerical variables.

Cytokine values were compared between amelanotic oral melanoma and melanotic oral melanoma expressed at the time of surgery using the Mann–Whitney U test. Friedman’s non-parametric test was performed to explore if there were significant differences over different time points. Mean comparisons between the groups (absence of the disease, presence of the tumor, and metastasis) were evaluated using the Mann–Whitney U test or the Kruskal–Wallis test when appropriate.

The statistical program R was used for statistical analysis (R version 4.3.2).

## 3. Results

Animals and serum samples. The main characteristics of the affected dogs are depicted in Table 1. The collected data includes breed, sex, age, reproductive state, diagnosis, TNM classification [16], and tumor size. Among the 10 oral melanomas measured on the day of surgical excision, 1 case had a maximum dimension of less than 2 cm, 8 cases ranged from 2 to 4 cm, and 1 case exceeded 4 cm, regardless of the stage. Histological examination of the excision margins revealed that all the dogs had non-infiltrated margins. Additionally, the mitotic index was found to be ≥4/10 high-power fields (HPF) in all dogs.

Out of a total of 10 patients, 5 remained alive until the completion of the 1-year follow-up, and 5 died due to metastasis (Table 2). In total, 31 serum samples (Table 3) were collected and analyzed, including 10 from patients with OMM on the day of surgery (T0) and 21 from follow-ups (T1, T2.1, T2.2). Of these, 16 were from patients in remission, 2 from those with OMM recurrence, and 3 from patients with metastasis, with 2 patients dying before their scheduled blood extraction. Samples were collected until patients completed 1 year of follow-up or died, with T0 indicating OMM presence, T1 representing the first 4 months post-tumor removal, and T2.1 and T2.2 referring to samples taken every 4 months until the conclusion of the one-year follow-up.

**Figure 1:**
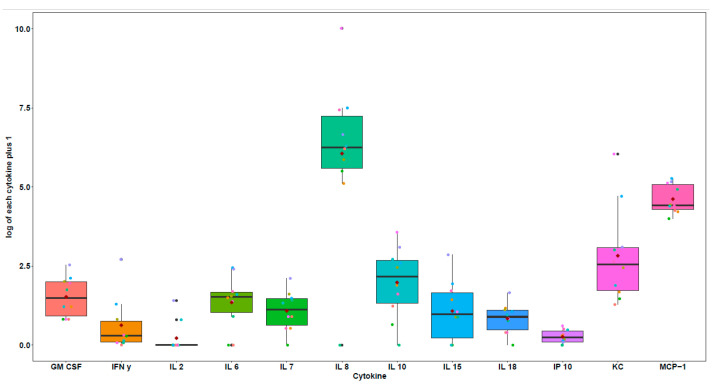
Cytokines at time 0 (T0), OMM samples.

Cytokines evaluation and follow-up. The samples were categorized as OMM-diseased dog samples (Figure 1) and revision samples, which included samples from patients with metastasis and samples from OMM dogs in remission.

**Figure 2:**
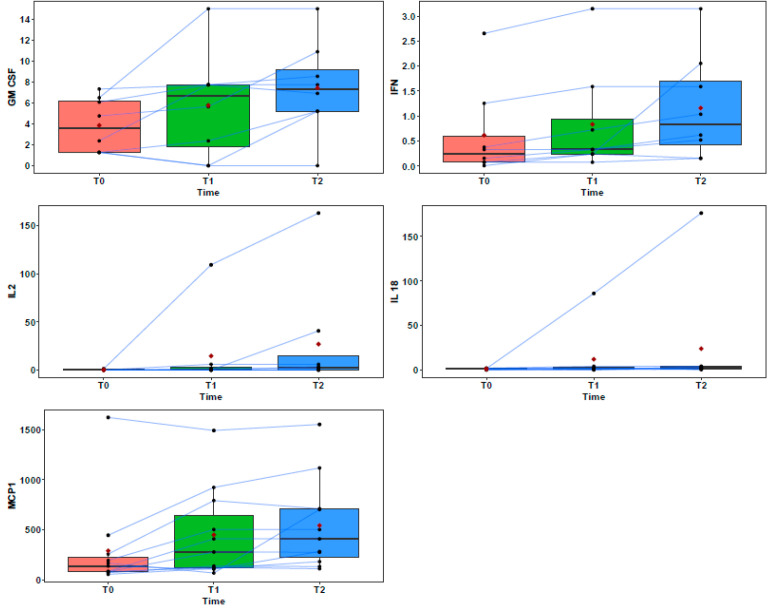
Significant differences between cytokines over time. This figure displays cytokine concentrations using box plots. The central line in each box indicates the median, the boxes show the interquartile range (IQR), and the whiskers represent the range excluding outliers. The red dots represent the mean value for each time point, and the connecting lines trace the individual trajectories of each dog across time.

Pairwise comparisons indicate that there were significant higher serum cytokine levels between the T2 and initial T0 values for GM-CSF (7.31 pg/mL and 3.56 pg/mL; *p* = 0.043), IFN-γ (0.83 pg/mL and 0.24 pg/mL; *p* = 0.002), MCP-1 (408.55 pg/mL and 136.49 pg/mL; *p* = 0.024), IL-2 (2.64 pg/mL and 0 pg/mL; *p* = 0.014) and IL-18 (2.46 pg/mL and 1.23 pg/mL; *p* = 0.04). Figure 2 shows the GM-CSF, IFN-γ, MCP-1, IL-2, and IL-18 values over time.

When the patients were evaluated individually, it was observed that, although not statistically significant, the serum levels of GM-CSF, IFN-γ, IL-2, and IL-18 were negatively associated with the patients who presented with disease recurrence and/or metastasis. In contrast, MCP-1 increased in all the revision samples, with particularly elevated levels observed in the metastatic samples (Table 4, Table 5, Table 6, Table 7 and Table 8).

**Figure 3:**
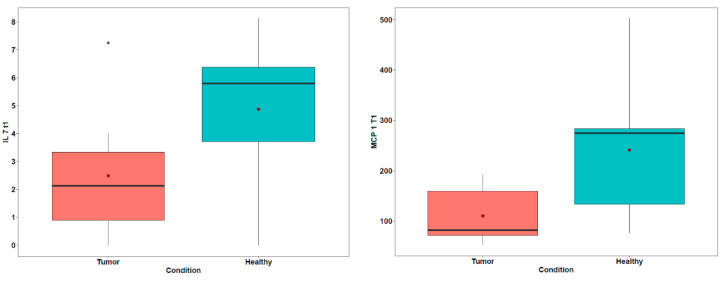
Comparison of cytokine concentrations between the OMM samples and the OMM dogs in remission samples. This figure presents the mean cytokine concentrations over time, with shaded areas representing the standard deviation.

Evaluation of variability in different sample groups. To examine whether there were significant differences between the OMM-diseased dogs samples, the OMM-metastasis dogs samples, and the OMM dogs in remission samples, we first compared the cytokine concentrations between the OMM dogs in remission and the OMM-diseased dogs samples. IL-7 and MCP-1 increased in patients who were in remission after one year of follow-up (5.8 pg/mL and 2.12 pg/mL, *p* = 0.078; 274.68 pg/mL and 81.92 pg/mL, *p* = 0.051, respectively; Table 9, Figure 3). Although both differences did not result in the threshold value of significance, which is a *p* value < 0.05, the small sample size should be taken into consideration, with the statistical analysis showing *p* values between 0.05 to 0.1, supporting a trend towards significance [17,18].

To explore the serum biomarkers predictive of clinical outcome among dogs with OMM, the OMM-diseased dogs samples were compared with the OMM-metastasis samples. The results showed that the dogs with OMM metastasis displayed higher serum MCP-1 than the OMM-diseased dogs (702.88 pg/mL and 136.49 pg/mL, *p* = 0.036, respectively; Table 10). There were no significant associations between the other serum factors and clinical prognosis.

There were no significant differences between the OMM-metastasis samples and the OMM dogs in remission samples.

Study of variability among OMM subtypes. Consistent with previous studies demonstrating biological differences between amelanotic tumor and melanotic tumor, comparisons were performed between six amelanotic and four melanotic OMM. IL-6, IL-10, and IL-15 concentrations showed a trend towards higher levels in the amelanotic OMM samples (4.22 pg/mL and 0.74 pg/mL, *p* = 0.042; 13.21 pg/mL and 1.67 pg/mL, *p* = 0.038; 3.89 pg/mL and 0.71 pg/mL, *p* = 0.066, respectively; Table 11).

Blood leukocyte analysis. The neutrophil-to-lymphocyte ratio (NLR) showed a moderate inverse correlation with the survival time in OMM patients (*p*-value = 0.033). Furthermore, Cox regression analysis between the NEU/LYM ratio outcomes indicated that a higher ratio is a risk factor for mortality (Table 12).

## 4. Discussion

This study highlights the significant potential of using canine oral malignant melanoma (OMM) as a model for understanding the mechanisms of immune evasion and developing novel immunotherapeutic strategies that could be relevant to both veterinary and human medicine [9]. Given the histopathological and clinical similarities between canine and human malignant melanomas [19,20,21], the findings provide valuable insights into the immunological dynamics at play during tumor progression and post-treatment.

Our findings demonstrate a significant increase in cytokines such as IL-2, IL-18, GM-CSF, IFN-γ, and MCP-1 following tumor removal. This suggests an activation of the immune response that may contribute to the antitumor effects observed in the post-surgical phase. Notably, high serum IL-2 levels were associated with prolonged survival, underscoring its crucial role in the immune response against melanoma [22,23,24]. Additionally, patients with prolonged overall survival exhibited high serum concentrations of GM-CSF, while those with recurrence of OMM and/or metastasis showed significantly lower levels of this cytokine. Consistent with previous studies [12,25], both IL-2 and GM-CSF appear to play a role in significantly increasing survival time. IFN-γ is a key mediator in enabling the immune system to recognize and control tumor development. Consequently, the impaired immune activity commonly observed in melanoma patients may facilitate immune evasion by tumor cells, promoting their proliferation and contributing to disease progression [26]. This coincides with our study, in which an increase in this cytokine can be observed once the tumor has been removed. Contrary to previous research suggesting that the presence of tumor cells or tumor-related stimuli could stimulate IL-18 production [27], our findings suggest that high concentrations play an antitumor role, and their absence predisposes patients to develop disease. MCP-1 also showed elevated levels post-surgery, although its dual role was evident—being associated with both positive outcomes and metastasis in different contexts [28,29,30,31,32]. This highlights the complex nature of MCP-1’s involvement in tumor dynamics. A possible explanation may relate to the involvement of MCP-1 in chronic inflammation or immune regulation, which could persist or even intensify during remission or advanced disease stages. However, we acknowledge that further studies are needed to fully understand the biological significance of this pattern.

The potential of IL-7 as a predictor of survival and a therapeutic agent [33,34] emerges as a significant finding in this study. Its role in enhancing immune responses suggests that it could be an important target for future therapeutic interventions [35,36,37]. On the other hand, MCP-1, while showing promise in some contexts, requires more in-depth exploration to fully understand its contribution to OMM progression and metastasis. The complex behavior of MCP-1 suggests that it could both inhibit and promote tumor growth [29,30,31,32] depending on the tumor microenvironment and stage of disease.

This study also underscores the biological differences between melanotic and amelanotic OMM [38], with the latter exhibiting higher levels of IL-6, IL-10, and IL-15. These findings reinforce the hypothesis that melanotic and amelanotic melanomas may behave differently, although no significant differences in aggressiveness were observed during follow-up. Understanding these differences is crucial for developing more targeted and effective therapies that consider the unique biological characteristics of each tumor subtype.

The neutrophil-to-lymphocyte ratio (NLR) is an inexpensive marker derived from basic blood tests. Given the interest in new inexpensive and simple predictive tools, blood NLR has demonstrated diagnostic and prognostic potential in human patients with inflammatory and neoplastic diseases [39,40,41,42]. This study also evaluated the diagnostic potential of the NLR in canine OMM and found the NLR to be significantly higher in patients who experienced shorter survival times.

This prospective study acknowledges its small sample size, primarily due to the reliance on dog owners’ commitment to attend scheduled follow-up visits, with 60% of dogs dying due to OMM-disease before the 1-year follow-up. Despite this limitation, we believe the findings hold significant value for guiding future research in this area.

Despite the inherent limitations in statistical power, canine clinical trials provide important insights into drug efficacy, safety, and resistance, and allow for faster data acquisition compared to human studies.

Prospective, randomized, double-blind clinical trials, free from confounding factors and reported according to established guidelines, are needed to enable meaningful comparisons and treatment recommendations for canine oral malignant melanoma (OMM). Additionally, increasing the sample size would help improve the statistical power and reliability of the findings.

### Future Directions

The identification and understanding of serum biomarkers in OMM could greatly enhance disease staging and prognosis, leading to more personalized treatment strategies. Continued research into these biomarkers is essential for improving survival rates and patient quality of life. This study contributes to the growing body of evidence that canine models can offer valuable insights that may also translate into more effective and targeted treatments for human melanoma.

## Figures and Tables

**Table 1:** Data collection.

Pt	Breed	Sex	Age (y)	Reproductive State	Diagnosis	TNM	Tumor Size (cm)
1	American staffordshire	F	10	Spayed	Oral melanoma	Stage II	2
2	Beagle	F	13	Spayed	Oral amelanotic melanoma	Stage III	3.2
3	Cocker spaniel	M	13	Spayed	Oral melanoma	Stage III	2.5
4	Labrador retriever	M	14	Spayed	Oral melanoma	Stage II	2
5	Mixed	F	12	Spayed	Oral melanoma	Stage I	1.5
6	Presa canario	M	9	Intact	Oral amelanotic melanoma	Stage III	7.7
7	Yorkshire terrier	M	14	Intact	Oral amelanotic melanoma	Stage II	2.1
8	Yorkshire terrier	M	12	Spayed	Oral amelanotic melanoma	Stage II	2.8
9	Yorkshire terrier	F	14	Spayed	Oral amelanotic melanoma	Stage III	2.5
10	Yorkshire terrier	M	14	Spayed	Oral amelanotic melanoma	Stage II	2

Abbreviations. Pt: patient. F: female. M: male. y: years, TNM: tumor–node–metastasis classification.

**Table 2:** Survival time in days.

Pt	Diagnosis	Survival Time (days)
1	OMM	365
2	OAM	43
3	OMM	131
4	OMM	310
5	OMM	365
6	OAM	365
7	OAM	365
8	OAM	155
9	OAM	240
10	OAM	365

Abbreviations. OMM: Oral malignant melanoma. OAM: Oral amelanotic melanoma.

**Table 3:** Serum samples collected.

Pt	T0	T1	T2.1	T2.2
1	OMM	Remission	Remission	Remission
2	OMM			
3	OMM	Metastasis		
4	OMM	Remission	OMM	Metastasis
5	OMM	Remission	Remission	OMM
6	OMM	Remission	Remission	Remission
7	OMM	Remission	Remission	Remission
8	OMM			
9	OMM	Remission	Metastasis	
10	OMM	Remission	Remission	Remission

Abbreviations. T0: sample extraction on the day of surgery. T1: first sample extraction after tumor removal. T2.1 and T2.1: subsequent sample extractions. OMM: oral malignant melanoma diseased dogs.

**Table 4:** Evolution over time of the GM-CSF variable concentration in pg/mL.

Pt	C0	T0	C1	T1	C2	T2
1	OMM	1.264	Remission	2.367	Remission	5.190
2 *						
3	OMM	6.477	Metastasis	4.969		
4	OMM	1.264	Remission	0.000	OMM	0.000
5	OMM	4.747	Remission	5.625	Remission	10.868
6	OMM	2.367	Remission	7.717	Remission	8.523
7	OMM	7.308	Remission	7.717	Remission	7.717
8 *						
9	OMM	1.264	Remission	0.000	Metastasis	0.000
10	OMM	6.054	Remission	7.717	Remission	6.895

Abbreviations. C0: condition at the time of sample collection on the day of surgery (T0). C1–C2: condition of the subsequent sample collection (T1, T2). *: missing values.

**Table 5:** Evolution over time of the IFN-γ variable concentration in pg/mL.

Pt	C0	T0	C1	T1	C2	T2
1	OMM	0.007	Remission	0.240	Remission	0.154
2 *						
3	OMM	1.255	Metastasis	1.591		
4	OMM	0.330	Remission	0.330	OMM	0.620
5	OMM	0.075	Remission	0.240	Remission	2.055
6	OMM	0.154	Remission	0.330	Remission	0.521
7	OMM	2.655	Remission	3.149	Remission	3.149
8 *						
9	OMM	0.075	Remission	0.075	Metastasis	0.154
10	OMM	0.377	Remission	0.722	Remission	1.037

*: missing values.

**Table 6:** Evolution over time of the IL-2 variable concentration in pg/mL.

Pt	C0	T0	C1	T1	C2	T2
1	OMM	0.000	Remission	0.000	Remission	3.366
2 *						
3	OMM	0.00	Metastasis	5.930		
4	OMM	0.00	Remission	0.000	OMM	0.000
5	OMM	0.000	Remission	0.000	Remission	40.683
6	OMM	1.23	Remission	109.302	Remission	163.074
7	OMM	0.000	Remission	0.000	Remission	0.000
8 *						
9	OMM	0.000	Remission	0.000	Metastasis	0.000
10	OMM	0.000	Remission	1.583	Remission	1.911

*: missing values.

**Table 7:** Evolution over time of the IL-18 variable concentration in pg/mL.

Pt	C0	T0	C1	T1	C2	T2
1	OMM	0.490	Remission	1.019	Remission	2.869
2 *						
3	OMM	2.061	Metastasis	4.244		
4	OMM	0.000	Remission	0.256	OMM	1.304
5	OMM	1.160	Remission	0.055	Remission	3.892
6	OMM	1.600	Remission	86.041	Remission	176.235
7	OMM	1.905	Remission	2.061	Remission	2.061
8 *						
9	OMM	1.304	Remission	2.219	Metastasis	0.747
10	OMM	0.490	Remission	1.304	Remission	

*: missing values.

**Table 8:** Evolution over time of the MCP-1 variable concentration in pg/mL.

Pt	C0	T0	C1	T1	C2	T2
1	OMM	69.391	Remission	136.491	Remission	131.637
2 *						
3	OMM	83.387	Metastasis	408.552		
4	OMM	53.473	Remission	112.197	OMM	180.804
5	OMM	136.491	Remission	104.868	Remission	281.735
6	OMM	79.710	Remission	276.785	Remission	274.679
7	OMM	193.172	Remission	502.113	Remission	502.113
8 *						
9	OMM	166.879	Remission	66.819	Metastasis	702.880
10	OMM	80.457	Remission	123.636	Remission	109.529

*: missing values.

**Table 9:** Comparison between OMM samples, regardless of metastatic status, and samples from OMM dogs in remission.

Cytokines	OMM-P50 (pg/mL)	OMM P25-P75 (pg/mL)	DIR-P50 (pg/mL)	DIR P25-P75 (pg/mL)	*p*-Value
IL-7	2.12	0.89–3.33	5.8	3.71–6.38	0.078
MCP-1	81.92	71.97–159.28	274.68	134.08–283.85	0.051

Abbreviations. OMM: oral malignant melanoma. DIR: OMM dogs in remission. P25: percentile 25. P50: median. P75: percentile 75.

**Table 10:** Comparison between the OMM samples and OMM-metastasis samples.

Cytokines	OMMr-P50 (pg/mL)	OMMr P25-P75 (pg/mL)	MET-P50 (pg/mL)	MET P25-P75 (pg/mL)	*p*-Value
MCP-1	136.49	79.71–193.17	813.18	629.3–972.26	0.015

Abbreviations. OMM: oral malignant melanoma. MET: OMM-metastasis. P25: percentile 25. P50: median. P75: percentile 75.

**Table 11:** Comparison between the OMM subtype and cytokines.

Cytokines	Tumor	P50 (pg/mL)	P25–P75 (pg/mL)	Tumor	P50 (pg/mL)	P25-P75 (pg/mL)	*p*-Value
IL-6	A	4.22	3.59–8.66	M	0.74	0–2.04	0.042
IL-10	A	13.21	7.31–19.3	M	1.67	0.68–4.49	0.038
IL-15	A	3.89	2.2–5.61	M	0.71	0–1.42	0.066

Abbreviations. A: amelanotic tumor. M: melanotic tumor. P25: percentile 25. P50: median. P75: percentile 75.

**Table 12:** Neutrophil-to-lymphocyte ratio (NLR).

Pt	NEU (K/µL)	LYM (K/µL)	NLR
1	7.14	1.93	3.699
2	4.68	0.6	7.800
3	8.26	1.29	6.403
4	2.308	0.89	2.593
5 *		1.2	
6	8.73	3.21	2.719
7	13.53	1.76	7.687
8	7.07	1.4	5.050
9	5.8	1.79	3.240
10	8.27	2.34	3.534

Abbreviations. NEU: neutrophil. LYM: lymphocyte. *: missing values.

## Data Availability

The original contributions presented in this study are included in the article. Further inquiries can be directed to the corresponding author.
